# Diarrheagenic *Escherichia coli* in Costa Rican children: a 9-year retrospective study

**DOI:** 10.1186/s13104-019-4313-1

**Published:** 2019-05-28

**Authors:** Cristian Pérez-Corrales, Kevin Leandro-Sandí

**Affiliations:** 1 División de Diagnóstico Molecular, Laboratorio Clínico, Hospital Nacional de Niños “Dr. Carlos Sáenz Herrera”, Caja Costarricense del Seguro Social, San José, Costa Rica; 2 División de Microbiología, Laboratorio Clínico, Hospital Nacional de Niños “Dr. Carlos Sáenz Herrera”, Caja Costarricense del Seguro Social, San José, Costa Rica

**Keywords:** Diarrheagenic *Escherichia coli*, Children, Gastroenteritis

## Abstract

**Objectives:**

This study aimed to estimate diarrheagenic *Escherichia coli* (DEC) prevalence among pediatric patients with diarrhea at the Costa Rican National Children’s Hospital-Social Security Service (Hospital Nacional de Niños-Caja Costarricense del Seguro Social; HNN-CCSS). DEC variations with respect to rainfall, presence of coinfections, and DEC antimicrobial resistance were also investigated.

**Results:**

A retrospective observational study from January 2008 to December 2016 was conducted. A total of 12 247 gastroenteritis records were analyzed. Annual DEC prevalence ranged from 2.7% (2008) to 9.0% (2013). The most prevalent pathotypes were enteroaggregative *E. coli* (EAEC) [n = 189 (31%)], enteropathogenic *E. coli* (EPEC) [n = 145 (24%)] and enteroinvasive *E*. *coli* (EIEC) [n = 91 (15%)]. A reduction in the probability of EAEC gastroenteritis was detected as rainfall rose above 200 mm/mo. [(Generalized Additive Model (GAM), p = 0.04)]. Coinfections were observed mainly between EPEC and *Campylobacter* spp. (10%). Antimicrobial resistance occurred in 0.6%, 29%, and 42% of DEC for ciprofloxacin, trimethoprim/sulfamethoxazole, and ampicillin, respectively.

## Introduction

Annually, diarrheal diseases account for more than two million deaths worldwide. Of these, more than half a million correspond to children under 5 years of age [1]. Diarrhea is defined as an increase in water content, volume, or frequency of stools, or as the passage of three or more loose or liquid stools per day [1, 2]. The broad spectrum of etiological agents, clinical syndromes and the multiple routes of pathogen transmission, highlight the need for accurate diagnostic strategies, clinical management and public health control programs [2]. However, this approach is often challenging in developing countries.

Since 2005, the Costa Rican National Children´s Hospital –Social Security Service (Hospital Nacional de Niños-Caja Costarricense del Seguro Social; HNN-CCSS) hosts the only Costa Rican state-run laboratory that performs molecular testing for diarrheagenic *Escherichia coli* (DEC), identifying six well-known pathotypes: Enteropathogenic *E. coli* (EPEC), enterotoxigenic *E. coli* (ETEC), shiga-toxin producing *E. coli* (STEC), enteroinvasive *E. coli* (EIEC), enteroaggregative *E. coli* (EAEC), and diffusely adherent *E. coli* (DAEC). Moreover, infections provoked by mixed pathotypes of *E. coli* (MPEC) can be identified when genes belonging to different pathotypes are concurrently found in a given strain. In Costa Rica, a previous study showed a predominant circulation of EPEC and EIEC between 2005 and 2007 [3]. The possible interaction between DEC and rainfall remained unexplored in Costa Rica. The aim of this study was to evaluate DEC prevalence and its association with rainfall, co-infections and resistance to antimicrobials in pediatric patients who were treated at the HNN-CCSS over a 9-year period (2008–2016).

## Main text

### Methods

#### Data included in the study

All stool records suggestive of gastroenteritis (loose or liquid samples, with or without leukocytes) between January 2008 and December 2016 were extracted from HNN-CCSS’s laboratory information systems: Labcore^®^ (Rochem Biocare, Bogotá, Colombia) and Copernico^®^ (Biomérieux, Marcy L’étoile, France) and collated with Microsoft Excel 2016. Diarrheic samples from outpatients under 13 years of age living in the central region of the country were included. Only the first sample per diarrheal episode was considered for analysis. Rainfall records for the study period were obtained from manual and automated weather stations located in the central region of Costa Rica and were kindly provided by the Costa Rican National Meteorological Institute.

#### Laboratory workup

All stool samples were analyzed at the time of sampling by the laboratory staff of the hospital. The standard laboratory procedure remained, in essence, unaltered throughout the study period. Predominant growth of yellow lactose-fermenting or bluish non-fermenting colonies on Tergitol-7 agar (Thermo Scientific™ Oxoid™) were investigated as a potential case of DEC gastroenteritis. Suspicious colonies were analyzed through an endpoint multiplex-PCR, as previously described [3]. The assay targets genes *eae*A, *bfp*A (EPEC); *stx*1, *stx*2 (STEC); *ial* (EIEC); *lt, st* (ETEC); *aggR*, *astA* (EAEC); and *daaE* (DAEC). Biochemical identification of isolates and antimicrobial susceptibility tests were performed using Vitek 2^**®**^ (BioMérieux) and Clinical Laboratory Standards Institute (CLSI) breakpoints.

#### Statistical analyses

Descriptive statistics were used to estimate the frequency of pathogens, antimicrobial susceptibility and coinfections for the study period. Differences in the proportion of pathogens between years were ascertained through adjusted loglinear models [4]. Generalized additive models (GAM) with Poisson distribution were fitted to evaluate the relationship between rainfall and the number of cases per pathotype. The impact of rainfall, DEC pathotypes, and their interaction with diarrheal cases were evaluated using a GAM model with a binomial distribution. The models were adjusted if they showed overdispersion and validated through diagnostic graphs. The level of significance was set at 0.05 (p < 0.05). The statistical analyses were carried out with R version 3.4.1 [5]. The GAM models were adjusted with the MGCV [6] and MASS [7] packages, and the smoothed functions that were significant with the VISREG package [8] and GGPLOT2 [9] were plotted.

### Results

#### Database records

A total of 46 906 fecal samples from 2008 to 2016 were studied. Of these, 26% (n = 12 247) matched the definition criteria for diarrhea [2008 (n = 1068); 2009, (n = 1054); 2010, (n = 1301); 2011, (n = 1046); 2012, (n = 1319); 2013, (n = 1249); 2014, (n = 1341); 2015, (n = 1964); 2016, (n = 1905)] and were selected for further analysis.

#### DEC prevalence throughout 2008–2016

DEC cases and prevalence were distributed as follows: [2008 (n = 29; 2.7%); 2009 (n = 31; 2.9%); 2010 (n = 64; 4.9%); 2011 (n = 56; 5.4%); 2012 (n = 98; 7.4%); 2013 (n = 112; 9.0%); 2014 (n = 65; 4.8%); 2015 (n = 86; 4.4%); 2016 (n = 68; 3.6%)]. Significant differences in prevalence were observed throughout the study period (Log-linear regression (LR), *χ*^2^ = 84.2; p < 0.0001). Notably, 2013 showed a nearly 3-fold increase in DEC prevalence (9.0%) with respect to 2008, whereupon prevalence decreased to 3.6% (n = 68) by 2016.

#### Distribution of DEC pathotypes

**Fig. 1:**
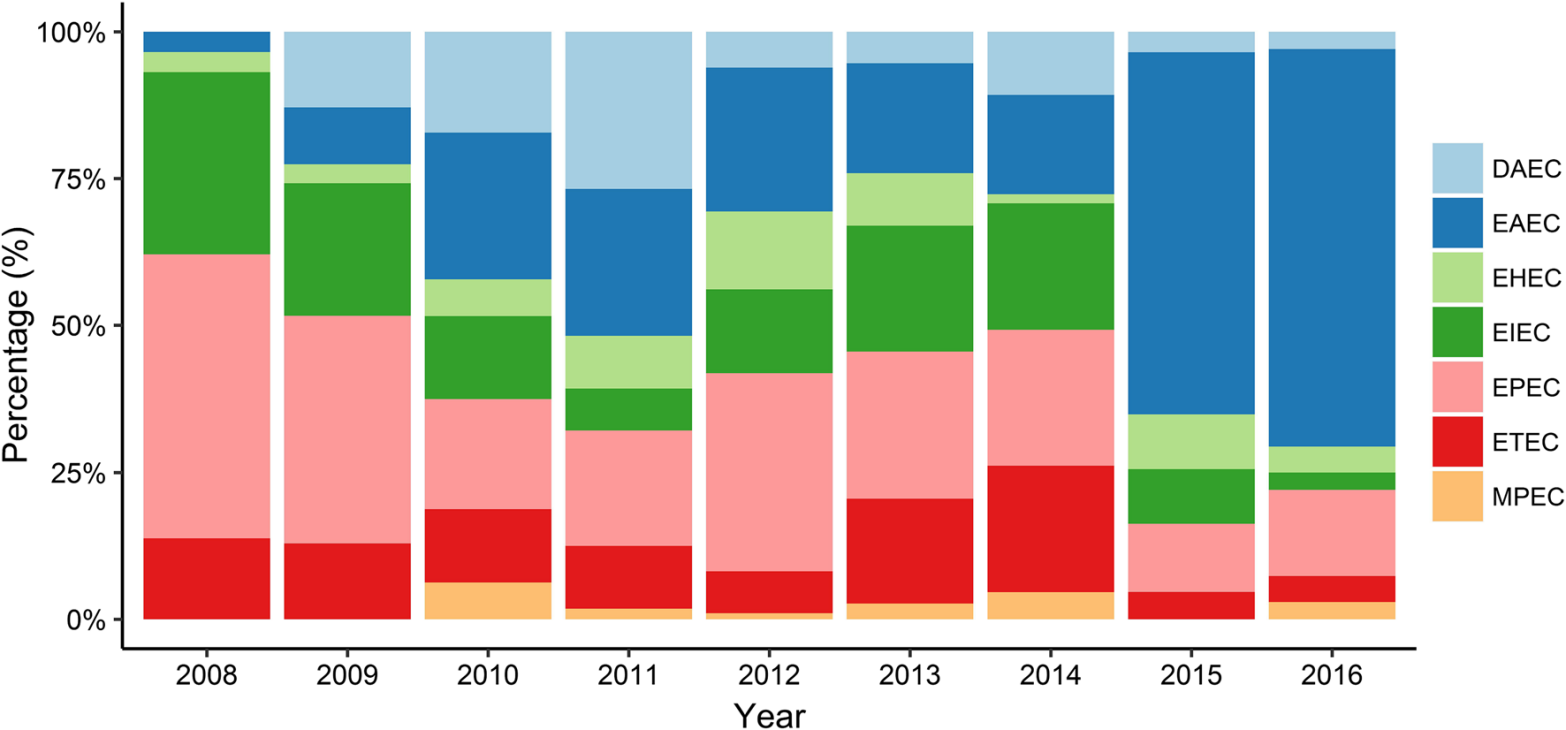
Distribution of DEC pathotypes from 2008 to 2016

The proportion of DEC pathotypes differed significantly over the years **(**LR, *χ*^2^ = 195.6; p < 0.0001**).** EAEC was the most frequent pathotype identified [31% (n = 189)], followed by EPEC [24% (n = 145)], EIEC [15% (n = 91)], ETEC [11% (n = 70)], DAEC [9% (n = 54)], STEC [8% (n = 46)], and MPEC [2% (n = 14)] (see Fig. 1).

#### DEC pathotypes and rainfall records

**Fig. 2:**
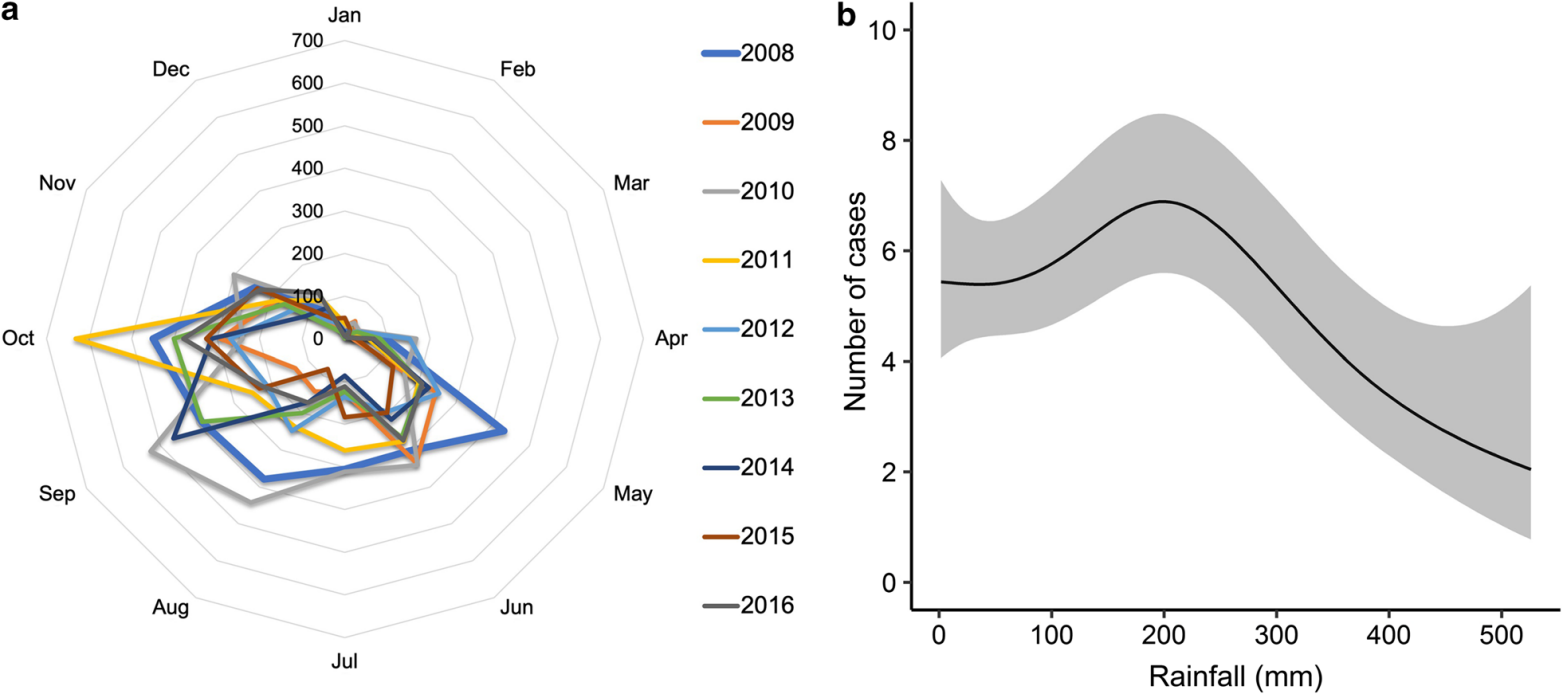
**a** Radial plot showing monthly average rainfall (mm) throughout the study period (2008–2016). **b** Relationship between cases of DEC diarrheal disease and monthly rainfall (2008–2016). The line represents the predicted smoothed function for DEC and the grey-shaded area corresponds to 95% point-wise confidence bands

Rainfall records depicted two well-defined seasons (dry and rainy), as well as noticeable variations in monthly rainfall averages between the years (Fig. 2a). The interaction between monthly rainfall and DEC cases was significant (GAM; *χ*^2^ = 33.22; p < 0.05). This interaction had a sigmoidal pattern, as shown in Fig. 2b.

**Fig. 3:**
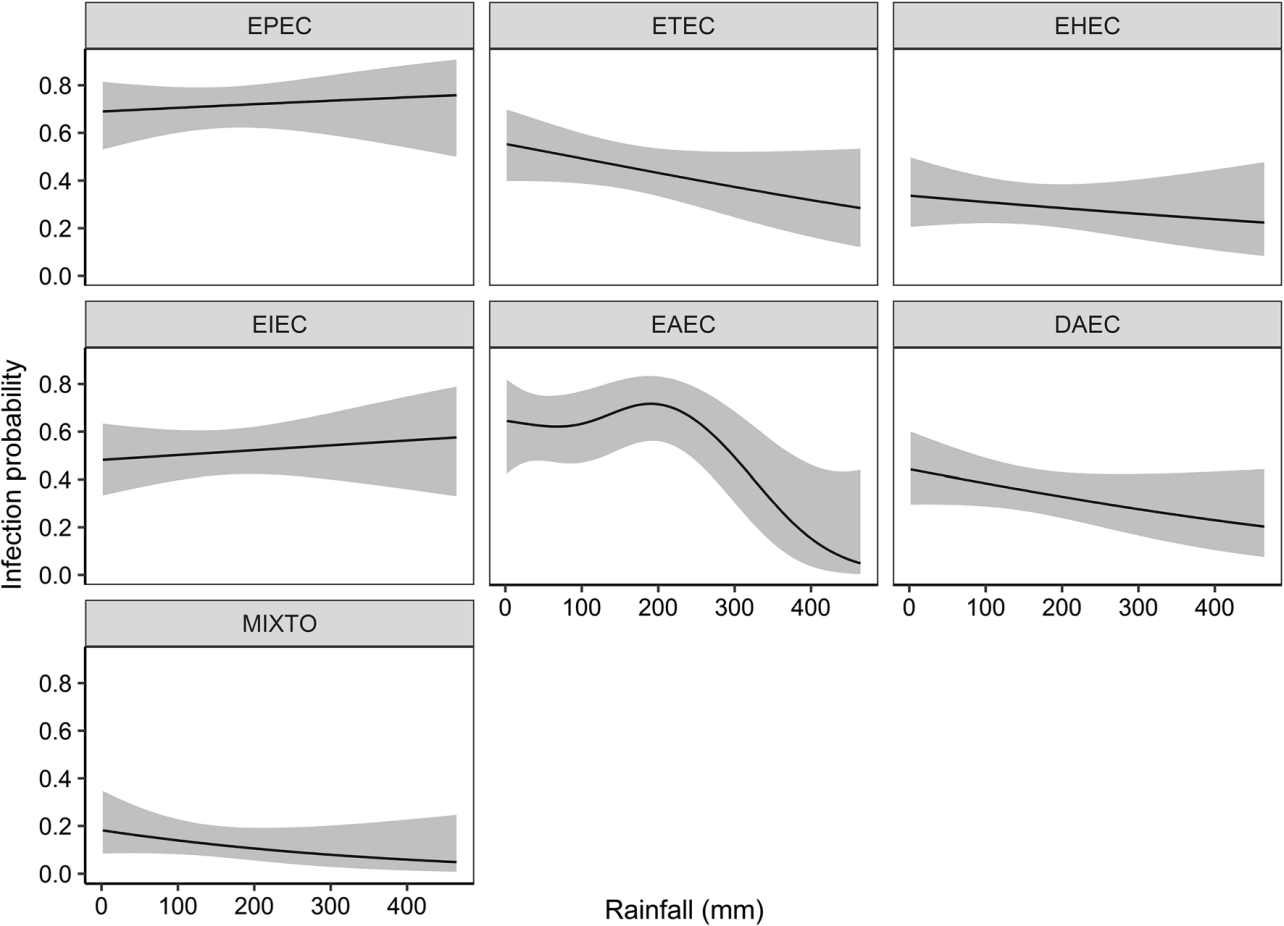
DEC infection probability changes and monthly rainfall (mm). The lines represent the predicted smoothed functions of DEC pathotypes and the grey-shaded area the 95% point-wise confidence bands

We also estimated the infection probability for each pathotype. Among the pathotypes investigated, only the EAEC infection probability and monthly rainfall was significant (Fig. 3).

#### Coinfections

Coinfections were found across most pathotypes, except for MPEC, and the global prevalence was estimated at 11% (n = 64). Frequency of co-infections ranged between pathotypes [EPEC 18% (n = 26), ETEC 13% (n = 9), EIEC 13% (n = 12), DAEC 11% (n = 6), EAEC 5% (n = 10), STEC 2% (n = 1)]. EPEC-*Campylobacter* coinfection was the most prevalent (10%; n = 14).

#### Antimicrobial susceptibility

Antimicrobial susceptibility to ampicillin (AMP), trimethoprim/sulfamethoxazole (TMP/STX) and ciprofloxacin (CIP) differed among DEC pathotypes. Approximately 25-30% of EPEC, ETEC, STEC and EIEC isolates showed resistance to AMP, whereas this proportion increased to 50% for MPEC, 54% for EAEC, and 77% for DAEC. TMP/STX resistance was found in 16% of EPEC and ETEC, 23.3% of STEC and EIEC, 33.9% of EAEC, 40% of MPEC, and 60% of DAEC. A generalized susceptibility to CIP was found across all DEC pathotypes. Over a span of 9 years, only one STEC isolate showed resistance to all three antimicrobials.

### Discussion

To estimate the burden of DEC gastroenteritis, we analyzed more than 46 906 laboratory records, of which only 26% (n = 12 247) fulfilled the inclusion criteria. DEC was identified in 609 (5.0%) of these samples. A similar study found DEC in 7.44% of cases in children under 5 years of age [10], whilst a small study found it in 6/155 (3.9%) of single infections using a molecular gastrointestinal panel [11]. We previously demonstrated the concurrent circulation of all DEC pathotypes and a predominance of EPEC and EIEC in a study from 2005 to 2007 [3]. The changing epidemiology of this pathogen has been described in different studies and geographical regions [12–18]. Importantly, this work provides evidence showing this phenomenon over an extended period of evaluation. This study unveiled a gradual shift in the contribution of specific pathotypes to DEC gastroenteritis, wherein the share of EAEC cases progressively increased as those attributable to EPEC declined. EAEC has been recently acknowledged as the leading DEC pathotype [13, 15, 19–23].

Regarding STEC, its prevalence remained low over the span of this study. Nonetheless, its association with large outbreaks, hemolytic uremic syndrome (HUS) and the high mortality rates emphasize the relevance of this pathotype for public health [24, 25]. To the best of our knowledge, asymptomatic STEC carriage in children [26] has not been studied thus far in Costa Rica, thereby hampering the formulation of policies to prevent its dissemination.

#### DEC and rainfall

The ubiquity of *E. coli* is well-documented and makes this bacterium a classic indicator of fecal contamination in water sources [27]. DEC presence in water used for human activities emphasizes the need to track not only the frequency of diarrheal disease but also the spatio-temporal interaction between the environment and this pathogen [28–30]. By virtue of the high variability of rainfall in tropical countries, we hereby described an innovative approach, analyzing DEC gastroenteritis in terms of monthly rainfall instead of seasonality. Using this approach, we found a marked drop in the EAEC infection probability and rainfall above 200 mm. We hypothesize that sudden rain showers and incipient rains heralding the rainy season might trigger EAEC diarrheal cases. In contrast, all other DEC pathotypes did not seem to interact with rainfall. Additional environmental and human factors might better predict the DEC infection probability by pathotypes other than EAEC.

#### Coinfections

We found 11% of coinfections between DEC and other enteropathogens. Changes in gut microbiota induced by DEC infection and the potential interaction with other pathogens have been reviewed elsewhere [31]. *Campylobacter* spp. was the most prevalent pathogen (4%) co-infecting pediatric patients in this study, which were predominantly associated with EPEC (10% of the cases) and ETEC (6% of the cases). The coinfection between EPEC and *Campylobacter* was also found at similar rates in a multicenter study in Europe, using a multiplex PCR-based approach [32].

#### Antimicrobial susceptibility

We found different antimicrobial susceptibility patterns among DEC pathotypes. Notably, 54% of EAEC isolates were mono-resistant to AMP, 34% to TMP/STX, and 33% showed resistance to both. Resistance to multiple antimicrobials is a major concern in EAEC [15], considering its high prevalence in children under 5 years of age and the possibility of asymptomatic carriage [33, 34]. Unlike the previous study [3] by Pérez et al. we did not find marked susceptibility to AMP in EPEC. Rather, a 25.6% resistance rate was observed, which was similar to ETEC (24%) and suggested increased rates of resistance in these two pathotypes. We hypothesize that this shift may be explained by a higher circulation, in recent years, of DEC strains carrying mobile genetic elements, which confer resistance to AMP. The rapid changes in local antibiotic susceptibility justify the active surveillance of DEC for epidemiological and public health purposes.

### Conclusions

For more than a decade, DEC has been investigated in stool specimens via molecular methods at the HNN-CCSS. Through this work, we are now aware of the local distribution, prevalence, antimicrobial resistance, and the interactions between each DEC pathotype and rainfall for most of this period.

Since March 2017, a syndromic multiplex PCR is part of the routine tests performed directly on diarrheic stool specimens for the simultaneous detection of multiple pathogens. This novel molecular approach will improve diagnostic efficiency through reduced turnaround times and an extended coverage of agents targeted in a single assay, thereby enabling a timely management and the instauration of appropriate antimicrobial therapy when warranted. The overall impact of this strategy will require an exhaustive review, for which this study establishes a reference against which future research can draw upon to determine the influence of technical innovation on the epidemiological profile of infectious gastroenteritis at the HNN-CCSS.

## Limitations

It is possible that a minor fraction of data may be missing due to the retrospective nature of this study. Additionally, this is a hospital and laboratory-based study only. Consequently, it does not include all children diagnosed with DEC gastroenteritis in the central region of Costa Rica. It is likely that the actual DEC pediatric disease was greater than depicted in this study.

## Data Availability

The datasets used and analyzed in the current study are available from the corresponding author on reasonable request and upon authorization of the competing institutions.

## References

[1] WHO. Diarrhoeal disease. WHO. 2017. http://www.who.int/news-room/fact-sheets/detail/diarrhoeal-disease. Accessed 19 Aug 2018.

[2] Guerrant RL, Van Gilder T, Steiner TS, Thielman NM, Slutsker L, Tauxe RV (2001). Practice guidelines for the management of infectious diarrhea. Clin Infect Dis.

[3] Pérez C, Gómez-Duarte OG, Arias ML (2010). Diarrheagenic *Escherichia coli* in Children from Costa Rica. Am J Trop Med Hyg.

[4] Bilder CR, Loughin TM. Analyzing a binary response, part 2: regression models. In: Analysis of categorical data with R. Boca Raton, FL: Chapman and Hall/CRC; 2014. p. 121–9.

[5] R Development Core Team RFFSC. R: A Language and Environment for Statistical Computing. R Foundation for Statistical Computing. 2008.

[6] Wood SN. GAMs in Practice: mgcv. In: Generalized additive models: an introduction with R. 2nd ed. New York: Chapman and Hall/CRC; 2017. p. 80.

[7] Venables WN, Ripley BD (2002). Modern applied statistics with S.

[8] Breheny P, Burchett W (2017). Visualizing regression models using visreg. R J..

[9] Wickham H (2009). ggplot2: elegant graphics for data analysis.

[10] Gómez-Duarte OG, Romero-Herazo YC, Paez-Canro CZ, Eslava-Schmalbach JH, Arzuza O (2013). Enterotoxigenic *Escherichia coli* associated with childhood diarrhoea in Colombia, South America. J Infect Dev Ctries..

[11] López-Medina E, Parra B, Dávalos DM, López P, Villamarín E, Pelaez M (2018). Acute gastroenteritis in a pediatric population from Cali, Colombia in the post rotavirus vaccine era. Int J Infect Dis..

[12] Torres AG (2017). *Escherichia coli* diseases in Latin America-a “One Health” multidisciplinary approach. Pathog Dis..

[13] Imdad Aamer, Foster Monique A., Iqbal Junaid, Fonnesbeck Christopher, Payne Daniel C., Zhang Chengxian, Chappell James D., Halasa Natasha, Gómez-Duarte Oscar G. (2018). Diarrheagenic Escherichia coli and Acute Gastroenteritis in Children in Davidson County, Tennessee, United States. The Pediatric Infectious Disease Journal.

[14] Weiler N, Orrego M, Alvarez M, Huber C (2017). Detección molecular de *Escherichia coli* diarreogénica en pacientes pediátricos con síndrome diarreico agudo en Paraguay. Mem Inst Investig Cienc Salud..

[15] Canizalez-Roman A, Flores-Villaseñor HM, Gonzalez-Nuñez E, Velazquez-Roman J, Vidal JE, Muro-Amador S (2016). Surveillance of diarrheagenic *Escherichia coli* strains isolated from diarrhea cases from children, adults and elderly at Northwest of Mexico. Front Microbiol..

[16] Rivas M, Padola NL, Lucchesi PM, Masana M, Torres AG (2010). Diarrheagenic *Escherichia coli* in Argentina. Pathogenic *Escherichia coli* in Latin America.

[17] Vilchez S, Reyes D, Paniagua M, Bucardo F, Möllby R, Weintraub A (2009). Prevalence of diarrhoeagenic *Escherichia coli* in children from León, Nicaragua. J Med Microbiol.

[18] Spano LC, da Cunha KF, Monfardini MV, de Cássia Bergamaschi Fonseca R, Scaletsky ICA (2017). High prevalence of diarrheagenic *Escherichia coli* carrying toxin-encoding genes isolated from children and adults in southeastern Brazil. BMC Infect Dis.

[19] Lozer DM, Souza TB, Monfardini MV, Vicentini F, Kitagawa SS, Scaletsky ICA (2013). Genotypic and phenotypic analysis of diarrheagenic *Escherichia coli* strains isolated from Brazilian children living in low socioeconomic level communities. BMC Infect Dis.

[20] Dutta S, Guin S, Ghosh S, Pazhani GP, Rajendran K, Bhattacharya MK (2013). Trends in the prevalence of diarrheagenic *Escherichia coli* among hospitalized diarrheal patients in Kolkata, India. PLoS One..

[21] Ali MMM, Mohamed ZK, Klena JD, Ahmed SF, Moussa TAA, Ghenghesh KS (2012). Molecular characterization of diarrheagenic *Escherichia coli* from Libya. Am J Trop Med Hyg..

[22] Garcia PG, Silva VL, Diniz CG (2011). Occurrence and antimicrobial drug susceptibility patterns of commensal and diarrheagenic *Escherichia coli* in fecal microbiota from children with and without acute diarrhea. J Microbiol..

[23] Somda NS, Bonkoungou OJI, Zongo C, Kpoda DS, Tapsoba F, Bassolé IHN (2017). Prevalence of *Escherichia coli* virulence genes in patients with diarrhoea in Ouagadougou, Burkina Faso. Afr J Clin Exp Microbiol..

[24] Paton JC, Paton AW (1998). Pathogenesis and Diagnosis of Shiga Toxin-Producing *Escherichia coli* Infections. Clin Microbiol Rev.

[25] Nataro James P., Kaper James B. (1998). Diarrheagenic Escherichia coli. Clinical Microbiology Reviews.

[26] Harries M, Dreesman J, Rettenbacher-Riefler S, Mertens E (2016). Faecal carriage of extended-spectrum β-lactamase-producing Enterobacteriaceae and Shiga toxin-producing *Escherichia coli* in asymptomatic nursery children in Lower Saxony (Germany), 2014. Epidemiol Infect.

[27] Edberg SC, Rice EW, Karlin RJ, Allen MJ (2000). *Escherichia coli*: the best biological drinking water indicator for public health protection. J Appl Microbiol.

[28] Ramírez Castillo FY, Avelar González FJ, Garneau P, Márquez Díaz F, Guerrero Barrera AL, Harel J (2013). Presence of multi-drug resistant pathogenic *Escherichia coli* in the San Pedro River located in the State of Aguascalientes, Mexico. Front Microbiol..

[29] Estrada-Garcia T., Lopez-Saucedo C., Thompson-Bonilla R., Abonce M., Lopez-Hernandez D., Santos J. I., Rosado J. L., DuPont H. L., Long K. Z. (2008). Association of Diarrheagenic Escherichia coli Pathotypes with Infection and Diarrhea among Mexican Children and Association of Atypical Enteropathogenic E. coli with Acute Diarrhea. Journal of Clinical Microbiology.

[30] Paredes-Paredes M, Okhuysen PC, Flores J, Mohamed JA, Padda RS, Gonzalez-Estrada A (2011). Seasonality of diarrheagenic *Escherichia coli* pathotypes in the US students acquiring diarrhea in Mexico. J Travel Med..

[31] Gallardo P, Izquierdo M, Vidal RM, Chamorro-Veloso N, Rosselló-Móra R, O’Ryan M (2017). Distinctive gut microbiota is associated with diarrheagenic *Escherichia coli* infections in chilean children. Front Cell Infect Microbiol..

[32] Spina A, Kerr KG, Cormican M, Barbut F, Eigentler A, Zerva L (2015). Spectrum of enteropathogens detected by the FilmArray GI panel in a multicentre study of community-acquired gastroenteritis. Clin Microbiol Infect.

[33] Amaya Erick, Vilchez Samuel, Paniagua Margarita, Möllby Roland, Nord Carl Erik, Weintraub Andrej, Reyes Daniel (2011). Antibiotic resistance patterns of intestinal Escherichia coli isolates from Nicaraguan children. Journal of Medical Microbiology.

[34] Hebbelstrup Jensen B, Olsen KEP, Struve C, Krogfelt KA, Petersen AM (2014). Epidemiology and clinical manifestations of enteroaggregative *Escherichia coli*. Clin Microbiol Rev.

